# Antiseptics

**Published:** 1893-05

**Authors:** 


### Antiseptics.

There would appear to be some hope still left for our old-fashioned, but somewhat neglected, friend, carbolic acid. Doubtless some practitioners have continued to use it, in spite of the inducements to try new germicides, due to the strong praise given to corrosive sublimate and other drugs. At all events, so far as surgical dressings are concerned, carbolic acid is likely to receive renewed attention on account of the reference made, as to its utility, by Sir Joseph Lister, in a recent post-graduate lecture at King's College Hospital. It was stated that, relying upon healthy blood-serum and the action of phagocytes, the surgeon might practically disregard the ordinary microbes of air-dust, and confine his attention to the grosser forms found on dirty sponges, instruments, and skin. Sir Joseph Lister expressed the opinion that Prof. Koch—able as he was—had been misled, and exaggerated the germicidal powers of corrosive sublimate. This corroborates one of the conclusions drawn in an American communication upon the subject, an abstract of which was published in our last issue. The lecturer recommended the use of gauze, steeped in a solution of carbolic acid of 1 to 20. This should afterwards be dusted over uniformly with cyanide of mercury and zinc.—*British Journnl of Dental Science.*

